# Individual behavioral variation reflects personality divergence in the upcoming model organism *Nothobranchius furzeri*

**DOI:** 10.1002/ece3.4356

**Published:** 2018-07-25

**Authors:** Eli S. J. Thoré, Laure Steenaerts, Charlotte Philippe, Arnout Grégoir, Luc Brendonck, Tom Pinceel

**Affiliations:** ^1^ Animal Ecology, Global Change and Sustainable Development KU Leuven Leuven Belgium; ^2^ Systemic Physiological and Ecotoxicological Research University of Antwerp Antwerp Belgium; ^3^ Water Research Group Unit for Environmental Sciences and Management North‐West University Potchefstroom South Africa; ^4^ Centre for Environmental Management University of the Free State Bloemfontein South Africa

**Keywords:** animal personality, behavioral ecology, behavioral variation, *Nothobranchius*, repeatability

## Abstract

In the animal kingdom, behavioral variation among individuals has often been reported. However, stable among‐individual differences along a behavioral continuum—reflective of personality variation—have only recently become a key target of research. While a vast body of descriptive literature exists on animal personality, hypothesis‐driven quantitative studies are largely deficient. One of the main constraints to advance the field is the lack of suitable model organisms. Here, we explore whether *N. furzeri* could be a valuable model to bridge descriptive and hypothesis‐driven research to further unravel the causes, function and evolution of animal personality. As a first step toward this end, we perform a common garden laboratory experiment to examine if behavioral variation in the turquoise killifish *Nothobranchius furzeri* reflects personality divergence. Furthermore, we explore if multiple behavioral traits are correlated. We deliver “proof of principle” of personality variation among *N. furzeri* individuals in multiple behavioral traits. Because of the vast body of available genomic and physiological information, the well‐characterized ecological background and an exceptionally short life cycle, *N. furzeri is* an excellent model organism to further elucidate the causes and implications of behavioral variation in an eco‐evolutionary context.

## 1 INTRODUCTION

Behavioral variation among individuals has been reported in a wide range of animals. Traditionally, such variation has been explained as the result of random stochastic variation (Briffa & Weiss, 2010; Tran & Gerlai, 2013) and behavior was assumed to be indefinitely plastic in response to environmental cues (Sih, Bell, & Chadwick, 2004). However, the finding that behavioral variation could be adaptive and is often consistent, with stable individual differences along a behavioral continuum, caused a shift in that classic interpretation and fueled the introduction of the term “animal personality” (Briffa & Weiss, 2010; Sih et al., 2004). Animal personality has since been demonstrated in an array of animals, ranging from mammals (Réale, Gallant, Leblanc, & Festa‐Bianchet, 2000), to birds (Drent, van Oers, & van Noordwijk, 2002), reptiles (Le Galliard, Paquet, Cisel, & Montes‐Poloni, 2013), amphibians (Wilson & Krause, 2012), fish (Adriaenssens & Johnsson, 2008), and invertebrates (Biro, Adriaenssens, & Sampson, 2014). There is growing awareness that animal personality should be considered to fully understand the ecology and evolutionary biology of species and can have major implications for ecosystem functioning (Mittelbach, Ballew, & Kjelvik, 2014; Roche, Careau, & Binning, 2016). Personality has been shown to influence an array of ecological and evolutionary aspects, including distribution patterns, feeding niche, population growth and persistence, migration, dispersal, species interactions, social evolution, and adaptive potential (Briffa & Weiss, 2010; Mittelbach et al., 2014; Wolf & Weissing, 2012). For instance, Ioannou, Payne, and Krause (2008) illustrated that bolder three‐spined stickleback (*Gasterosteus aculeatus*) preyed more heavily upon *Chironomus* prey, whereas Chapman et al. (2011) showed that bolder individuals of roach (*Rutilus rutilus*) had a higher tendency to migrate. The study of individual behavioral responses is, therefore, not only interesting from a fundamental perspective, but is also important for, for example, nature conservation, harvest and resource management as outlined in more detail by Mittelbach et al. (2014).

While a growing body of literature reports on animal personality in a variety of species, the ecological underpinnings that determine personality and its consequences remain poorly understood (Adriaenssens & Johnsson, 2017; Mittelbach et al., 2014). In addition, underlying proximate mechanisms (e.g. genetic, physiology) should be identified (Briffa, Sneddon, & Wilson, 2015; Oswald, Singer, & Robison, 2013) and animal personality should be studied across the ontogeny of organisms. This is a crucial step toward understanding the function, evolution, and mechanism of animal personality (Stamps & Groothuis, 2010). To tackle these goals, studies of individual behavioral variation in natural populations of well‐characterized species along with laboratory and mesocosm studies have been promoted (Killen et al., 2016; Mittelbach et al., 2014).

A number of fish model organisms are used in various fields of biological research (Polačik, Blažek, & Reichard, 2016). Best studied species include zebrafish (*Danio rerio*), medaka (*Oryzias latipes*), fathead minnow (*Pimephales promelas*), and stickleback (*Gasterosteus aculeatus*). One constraint with these models is their relatively slow maturation and long lifespan, hampering whole life or multigenerational studies (Harel et al., 2015). Annual killifish combine the perks of traditional fish models with the short generation time of invertebrate model species (Polačik et al., 2016). Still, in order to be used as models in personality research, the existence of consistent among‐individual variation in behavior needs to be demonstrated. Species of the African genus *Nothobranchius* inhabit temporary freshwater pools and are adapted to the seasonal desiccation of their habitat by completing their life cycle in typically 3–4 weeks (Cellerino, Valenzano, & Reichard, 2015; Polačik et al., 2016). Moreover, annual fish produce drought‐resistant, dormant eggs that form an egg bank in the sediment and hatch when the pool is inundated again (Reichard, Polačik, & Sedláček, 2009).

The African annual killifish *Nothobranchius furzeri* (Turquoise killifish) has the shortest lifespan of any vertebrate in captivity. The species typically matures after three weeks (both in laboratory and in field conditions) and survives for only 5–6 months post‐hatching (Blažek, Polačik, & Reichard, 2013; Terzibasi et al., 2008), with even shorter lifespans recorded for inbred (homozygous) lines (Polačik et al., 2016; Wang, Promislow, & Kaeberlein, 2015). The “fastest” fish in our breeding facility even reached maturity in a little under two weeks (E. Thoré, Personal observation). In addition to a very short lifespan and generation time, *N. furzeri* is relatively easy to culture (Polačik et al., 2016). Moreover, individuals typically have a high reproductive output and are naturally bold in behavior (Cellerino et al., 2015; Polačik et al., 2016). Dormant eggs can easily be stored and allow for a synchronized hatching for experiments (Philippe et al., 2017; Polačik et al., 2016).

*Nothobranchius furzeri* is a relatively novel, yet already widely used, model organism that is rapidly gaining popularity in many fields of biological research, including ecology (Grégoir et al., 2017; Grégoir et al., 2017; Pinceel et al., 2015; Reichard, Polačik, Blažek, & Vrtílek, 2014), evolutionary biology (Blažek et al., 2016), ecotoxicology (Philippe et al., 2017), gerontology (Reichwald et al., 2015), genome‐wide gene expression studies and quantitative genetics (Cellerino et al., 2015; Valenzano et al., 2015). This surge of interest has, for instance, resulted in the construction of a whole brain atlas (D'angelo, 2013), age‐related histopathological analyses, an annotated genome (Reichwald et al., 2015; Valenzano et al., 2015) and transcriptome (Di Cicco, Terzibasi Tozzini, Rossi, & Cellerino, 2011), transgenesis and the generation of transgenic lines (Hartmann & Englert, 2012; Valenzano, Sharp, & Brunet, 2011).

In this study, we explore and discuss how *N. furzeri* could be a valuable model organism to move from descriptive studies to quantitative personality research to further unravel the causes, function, and evolution of animal personality. Despite the magnitude of recent advances in a range of biological disciplines that continue to promote *N. furzeri* as a promising model organism, individual behavioral variation in *N. furzeri* remains to be examined. Consequently, it remains unknown whether behavioral differences among *N. furzeri* individuals reflect personality variation or are due to random stochastic variation. We study a range of behavioral measures in *N. furzeri* individuals to determine whether variation is reflective of stable individual differences or rather represents random stochastic variation as a first and necessary step for quantitative personality research. In addition, we examine if and how different behavioral traits are correlated with each other. As personality variation has already been confirmed in a range of fish species (Budaev & Brown, 2011), we expect stable individual differences in behavioral expression and correlated behaviors also to be present in *N. furzeri*.

## 2 METHODS

### 2.1 General setup and fish maintenance

A total of 20 *N. furzeri* fish (9 females, 11 males) were reared from egg to adulthood while quantifying a range of behavioral measures under common garden rearing conditions in the laboratory. Fish originate from the natural population MZCS‐414 (central Mozambique) and had been laboratory‐reared for three generations under optimal common garden conditions prior to the start of the experiment in accordance with the protocols as specified by Polačik et al. (2016). Fish were hatched (synchronized) by submerging eggs and peat in reconstituted water (type II RO water with added Instant Ocean salt mix; 8.3 pH, 600 μS/cm conductivity) at a temperature of 14°C, based on the protocol outlined by Polačik et al. (2016). Two days post‐hatching, fish larvae were transferred to housing tanks at a density of 20 larvae per 4‐L water. At an age of 2 weeks post‐hatching, fish were transferred to 10‐L aquaria in groups of 10. Three weeks post‐hatching and for the remainder of the experiment, fish were housed individually in 9‐L tanks for individual monitoring. Fish were placed in a housing compartment (one compartment per tank, approx. 12 cm L × 19 cm W × 16 cm H) to habituate them to the tank setup for behavioral testing (see below). Each compartment was provided with an air‐driven filter to ensure good water quality. The sides of the tanks were covered with opaque plastic partitions to prevent confounding social contact between individuals. If social contact was allowed, neighboring dominant males could have interacted more with each other and have higher energetic needs than would neighboring females or submissive males. Water was renewed every 2 days from the moment of hatching until 3 weeks post‐hatching. Afterward, water was renewed on a weekly basis. Fish larvae were fed an *ad libitum* quantity of *Artemia franciscana* nauplii (Ocean Nutrition, Essen, Belgium) twice a day until 3 weeks post‐hatching and frozen *Chironomus* larvae (Ocean Nutrition, Essen, Belgium), supplemented with *Artemia* nauplii, from an age of 3 weeks onward. On observation days, fish were fed only once a day to avoid interference with the observations. At an age of 6 weeks, a small amount of the solvent DMSO (dimethyl sulfoxide) was added to the water of the housing tanks as part of a concurrent experiment studying the effects of antidepressant exposure on behavioral expression in killifish. The applied solvent concentration was, however, negligible (0.00001 vol%) and identical for all individuals. Still, to account for any potential differences before/after DMSO addition, we included it as a random factor in our statistical analyses.

### 2.2 Behavioral setup

At an age of one month post‐hatching, individual fish were subjected to four different behavioral tests, each of which was repeated once per week for a total of five consecutive trials per test. For each trial, each fish was placed individually in an experimental arena and allowed to acclimate for 5 min prior to the start of the observation trial. After each behavioral trial, individuals were transferred back to their housing tanks. Behavioral tests included (a) an emergence test; (b) an open field test. To also include behavior with direct ecological relevance, a habitat choice test (c) and a life skills test (d) were included. For practical reasons and to minimize behavioral changes associated with timing of the day (Tran & Gerlai, 2013), each sampling burst per behavioral trial lasted for a maximum of 3.5 hr. Fish were divided in two observation cohorts for practical reasons, following the scheme presented in Table 1. In order to motivate fish to explore the arena and to prevent disinterest in the applied food, fish were abstained from food for 24 hr before the emergence test and life skills test.

**Table 1:** Weekly scheme of sampling bursts

Moment of the week	Cohort	Behavioral test
Tuesday		
Morning	2	Habitat choice test
Afternoon	1	Emergence test
Wednesday		
Morning	1	Habitat choice test
Afternoon	2	Emergence test
Thursday		
Morning	2	Open field test
Afternoon	1	Life skills test
Friday		
Morning	1	Open field test
Afternoon	2	Life skills test

Note

Every sampling burst lasted for a maximum of 3.5 hr. Fish were divided in two cohorts to improve the logistic feasibility of the experiment. Every Monday, medium of the housing tanks was renewed and there were no behavioral tests.

As body size can be an important covariate of behavioral expression (Polverino, Bierbach, Killen, Uusi‐Heikkilä, & Arlinghaus, 2016), it was determined on the day before the first behavioral observations by briefly transferring each individual to a petri dish with a small amount of water and taking size‐calibrated photographs (mean ± *SD* = 23.94 ± 1.70 mm). Measurements were performed using the open source image processing software ImageJ 1.50i (Schneider, Rasband, & Eliceiri, 2012).

### 2.3 Emergence test

**Figure 1:**
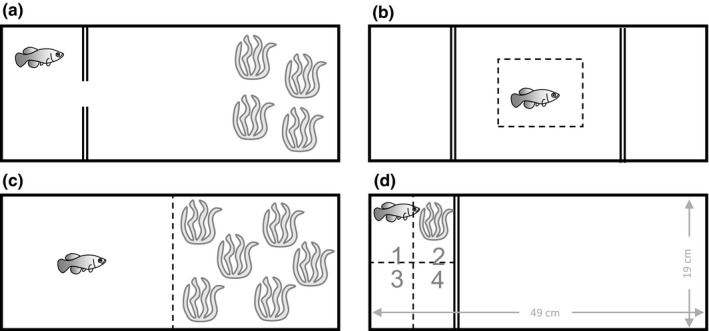
Schematic representation of the different test arenas used (top view). All tanks are LxWxH 49 × 19 × 16 cm and hold 9 L of water, except for the open field arena which only holds water to a height of 2 cm (approx. 1.9 L of water). (a) Experimental setup for the emergence test. The start compartment resembles the housing conditions. A doorway (diameter 20 mm) allows individuals to explore the novel, larger part of the tank which holds artificial plants as shelter in the furthest half of the compartment. (b) Open field experimental setup. (c) Experimental setup for the habitat choice test. The tank is equally divided in an open part and a part provided with artificial plants as shelter. The dotted line represents a virtual barrier. (d) Experimental setup for the life skills test, used to characterize feeding and antipredator behavior. The experimental compartment was virtually divided in four equally sized zones (delineated by the dotted lines). Zone 2 holds an artificial plant as shelter, whereas both feeding stimulus and simulated avian attack were applied in zone 3

Exploration tendency was studied by means of an emergence test. Individuals were transferred to an experimental arena (Figure 1a), similar to their housing tank (i.e. a smaller compartment, separated from a “novel” larger compartment). After the acclimatization period, a doorway was opened to allow the individual to leave the small compartment and explore the larger one. Latency time to enter the newly available compartment was recorded during the next 45 min. Fish that did not enter the compartment during this time were assigned the maximum score of 45 min.

### 2.4 Open field test

An open field test was performed to explore basic locomotor activity parameters and the propensity to take risks (boldness). Individuals were transferred to an open field arena (Figure 1b) and spontaneous activity was recorded for 20 min. The tank was virtually divided in a central (50% of length and width of open field) and peripheral area with activity in the centrum zone being considered as more risk‐prone than activity in the peripheral zone (Ansai, Hosokawa, Maegawa, & Kinoshita, 2016). Water level in this setup was lowered to a height of 2 cm to allow activity in the horizontal plane only.

### 2.5 Habitat choice test

The test arena to explore habitat choice was divided in two equal parts: a part with artificial plants for shelter and an open, barren part (Figure 1c). Fish were introduced individually to the centrum of the open part and habitat preference (expressed as the proportion of time spent in the open zone compared to the duration of the test) was recorded for 30 min after 5 min of acclimatization.

### 2.6 Life skills test

Feeding and antipredator behaviors were explored by means of a life skills test (Figure 1d). The experimental arena was virtually divided in four equal‐sized zones. After an acclimatization period, the test was initiated as soon as fish entered either zone 1 or 4, after which food (*Chironomus* larvae) was gently added in zone 3. The latency time to feed was assessed. At the onset of feeding, an avian predator attack was simulated by means of a suspended, weighted 15‐ml falcon tube (beak shaped, opaque) that was dropped and allowed to touch the water surface in zone 3 (see for instance Bell & Sih, 2007 and Hedgespeth, Nilsson, & Berglund, 2016 for a similar setup). Subsequently, the time until a fish that froze or fled in response to the simulated attack resumed movement and the time that was needed to resume feeding were recorded. Again, a maximum time of 45 min was allowed.

All behavioral measures were recorded (top view) using Logitech C920 HD Pro webcams and were manually analyzed afterward (observer‐blind), except for open field data which were analyzed using EthoVision XT Version 9.0 video‐tracking software (Noldus Information Technologies Inc; http://www.noldus.com).

### 2.7 Animal welfare note

This study was approved by the ethical committee of KU Leuven (file number: P160/2016). All performed procedures are conform the legal requirements for animal research in Belgium. The condition and health of every individual was checked multiple times a day by two researchers separately (ESJT and LS). In addition, water parameters were measured in each individual tank on a daily basis to keep track of water quality (pH: mean ± *SD* = 8.20 ± 0.41; conductivity: mean ± *SD* = 679.57 ± 22.64 μS/cm; temperature: mean ± *SD* = 24.55 ± 0.89°C). Animals were housed under optimal conditions and the handmade air‐driven filter provided shelter in all tanks. Disturbance and handling was kept to a minimum. Fish were part of a concurrent experiment studying the effects of antidepressant exposure on behavioral expression in killifish (only control fish were used for analysis in the current study). This approach enabled us to reduce the use of laboratory animals, by assessing multiple research objectives per experiment and avoiding the subsequent use of additional animals consistent with the “three Rs” guiding principles for more ethical use of laboratory animals (Fenwick, Griffin, & Gauthier, 2009).

### 2.8 Statistical analysis

All statistical analyses were conducted in R 3.3.1 (R Development Core Team, 2016) at a significance level of 0.05. Model assumptions were verified graphically for all analyses. Linear mixed models with Gaussian error distribution were fitted for all behavioral response variables (lme4 package; Bates et al., 2017) with sex, body size, and trial number (referring to the repeated measures, to account for behavioral changes over time) as fixed factors, including interaction between sex and body size and between sex and trial number. Fish identity was added to the model as random factor. To also account for variation explained by potential differences between cohorts and before/after DMSO treatment (0.00001 vol%), these factors were added to the models as random factors. Significance of fixed effects was tested using Wald chi‐square tests (car package; Fox et al., 2017). To determine if individual behavioral variation is reflective of personality variation, repeatability measures were calculated using the rptR package (Stoffel, Nakagawa, & Schielzeth, 2018) as the between‐individual variance over the sum of between‐individual and residual variance (Nakagawa & Schielzeth, 2010). Statistical significance of the repeatability measures was tested by likelihood‐ratio tests (comparing the model with and without the fish identity random effect structure) in the rptR package. Correlations between behavioral traits were assessed by averaging individual scores and calculating the Spearman correlation coefficient per pair of significantly repeatable behavioral traits.

### 2.9 Behavioral response variables

Latency time (in seconds) to enter the novel environment was assessed in the emergence test and was log‐transformed to meet model assumptions. Due to a low resolution in the data (36% did not emerge within the given 45 min and were assigned the maximum value), model assumption of homoscedasticity could not completely be met. Therefore, these results should be interpreted with some caution.

Total distance moved (cm) in the open field test was assessed as a measure of activity. As measures of boldness, the number of times the fish entered the centrum zone (log + 1 transformed) and the cumulative time (log + 1 transformed, in seconds) spent in the centrum zone were assessed.

Habitat choice (habitat choice test) was expressed as the proportion of time spent in the open zone compared to the total duration of the test (time spent in the open zone plus time spent in the plant zone).

Behavioral response variables for the life skills test included latency time (in seconds) to feed before and after the simulated predator attack and the time until movement (in seconds) after the attack for fish that froze or fled in response to the simulated attack. Latency time to feed before the predator attack was double log‐transformed to meet the model assumptions while latency time to resume feeding and time till movement after the predator attack were log‐transformed.

## 3 RESULTS

**Figure 2:**
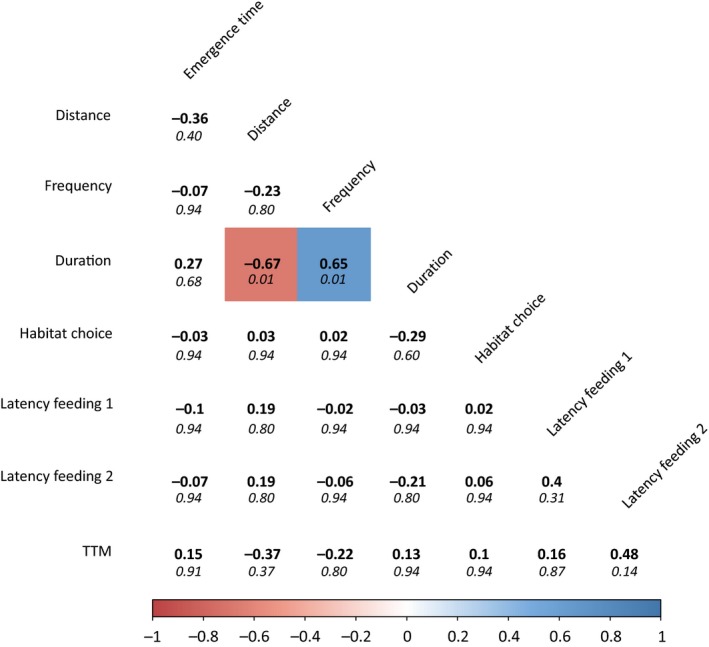
Spearman rank correlation coefficients (in bold) per pair of behavioral traits. *p*‐values (false discovery rate controlled) are shown in italics. Significant correlation coefficients are depicted in color (red for negative correlation, blue for positive correlation). Emergence time: latency time to enter novel environment (emergence test), Distance: total distance moved (open field test), Frequency: number of times the fish entered centrum (open field test), Duration: cumulative duration in centrum (open field test), Habitat choice: habitat preference (habitat choice test), Latency feeding 1: latency time to feed before attack (life skills test), Latency feeding 2: latency time to resume feeding (life skills test), TTM: time till movement after attack (life skills test)

All behavioral measures were repeatable. Latency to enter a novel environment in the emergence test was repeatable with *R* = 0.334 (*p* < 0.001). In the open field test, the total distance moved, the number of times the fish entered the centrum and the cumulative duration spent in the centrum were repeatable with *R* = 0.457 (*p* < 0.001), *R* = 0.266 (*p* < 0.001), and *R* = 0.259 (*p* = 0.002), respectively. Habitat choice was repeatable with *R* = 0.178 (*p* = 0.016). In the life skills test, latency time to feed before attack, latency time to resume feeding and time till movement after attack were repeatable with *R* = 0.174 (*p* = 0.012), *R* = 0.108 (*p* = 0.011), and *R* = 0.32 (*p* = 0.005), respectively. Mean value, standard deviation and minimum and maximum value for all behavioral response variables are presented in Table 2. Results of the mixed models are presented in Table 3. Correlation coefficients per pair of behavioral traits are presented in Figure 2.

**Table 2:** Mean value, standard deviation, and minimum and maximum value for all behavioral response variables, separated per sex. Latency time to initiate and resume feeding are expressed in seconds, as is latency time to resume movement after attack, latency time to enter novel environment, and cumulative time spent in centrum zone. Habitat choice was calculated as the total amount of time spent in the open zone (in seconds) over the total amount of time spent in the open and plant zone (i.e. duration of the test) and ranges between 0 (higher preference for plant zone) and 1 (higher preference for open zone). Total distance moved is expressed in centimeter

Behavioral response	Mean value		Standard deviation		Min. value		Max. value	
	Males	Females	Males	Females	Males	Females	Males	Females
Latency time to feed before attack (s)	24.462	109.744	43.261	299.682	2	3	200	1,597
Latency time to resume feeding (s)	155.173	277.878	308.888	486.678	3	5	1,679	2,296
Time till movement after attack (s)	17.526	14.353	24.669	17.029	1	2	123	80
Latency time to enter novel environment (s)	1404.077	1265.86	1083.338	993.898	36	42	2,700	2,700
Habitat choice	0.443	0.395	0.277	0.214	0	0.035	0.996	0.936
Total distance moved (cm)	2226.952	2119.377	910.573	891.153	661.78	679.55	3,892.33	4,306.76
Number of times entered in centrum	7.577	5.690	6.458	4.841	0	0	26	19
Cumulative duration in centrum (s)	38.005	25.371	44.046	21.787	0	0	197.21	95.9

**Table 3:** Results of the mixed models per behavioural measure

Behavioral response	Sex	Trial	Sex × Trial	Body size	Sex × Body size
Emergence test					
Latency time to enter novel environment	χ^2^ = 0.361	χ^2^ = 2.103	χ^2^ = 0.834	χ^2^ = 0.439	χ^2^ = 0.690
	*p* = 0.548	*p* = 0.147	*p* = 0.361	*p* = 0.508	*p* = 0.406
Open field test					
Total distance moved	χ^2^ * *< 0.001	χ^2^ = 9.152	χ^2^ = 1.712	χ^2^ = 0.108	χ^2^ = 3.187
	*p* = 0.992	*p* = **0.002**	*p* = 0.191	*p* = 0.743	*p* = 0.074
Number of times the fish entered centrum	χ^2^ = 1.287	χ^2^ = 0.047	χ^2^ = 1.748	χ^2^ = 0.472	χ^2^ = 3.250
	*p* = 0.257	*p* = 0.829	*p* = 0.186	*p* = 0.492	*p* = 0.071
Cumulative duration in centrum	χ^2^ = 1.586	χ^2^ = 1.214	χ^2^ = 1.370	χ^2^ = 2.130	χ^2^ = 2.896
	*p* = 0.208	*p* = 0.271	*p* = 0.242	*p* = 0.145	*p* = 0.089
Habitat choice test					
Habitat choice	χ^2^ * *< 0.001	χ^2^ = 1.651	χ^2^ = 0.615	χ^2^ = 0.535	χ^2^ = 0.030
	*p* = 0.985	*p* = 0.199	*p* = 0.433	*p* = 0.465	*p* = 0.863
Life skills test					
Latency time to feed before attack	χ^2^ = 1.305	χ^2^ = 0.623	χ^2^ = 3.291	χ^2^ = 0.047	χ^2^ = 0.047
	*p* = 0.253	*p* = 0.430	*p* = 0.070	*p* = 0.829	*p* = 0.829
Latency time to resume feeding	χ^2^ = 1.028	χ^2^ = 1.755	χ^2^ = 2.123	χ^2^ = 0.011	χ^2^ = 0.363
	*p* = 0.311	*p* = 0.185	*p* = 0.145	*p* = 0.918	*p* = 0.547
Time till movement after attack	χ^2^ = 3.107	χ^2^ = 0.165	χ^2^ = 9.451	χ^2^ = 2.145	χ^2^ = 0.154
	*p* = 0.078	*p* = 0.684	*p* = **0.002**	*p* = 0.143	*p* = 0.695

Note

*p*‐values <0.05 are shown in bold.

## 4 DISCUSSION

Individual behavioral variation is a common phenomenon in animals. Scientists now increasingly recognize the importance of decomposing such variation in among‐ and within‐individual variation and argue that consistent individual differences along a behavioral continuum exist (Briffa & Weiss, 2010; Roche et al., 2016; Sih et al., 2004). Here, we investigated if behavioral variation in the upcoming vertebrate model organism *N. furzeri* is reflective of stable individual differences or rather represents random stochastic variation. Overall, our results support consistent inter‐individual differences in multiple behavioral responses, suggestive of personality variation. All behavioral measures that reflect locomotor activity and the propensity to take risks were found to be repeatable. This suggests that consistent behavioral differences exist among *N. furzeri* individuals.

Locomotor activity and movement resumption after a simulated predator attack of *N. furzeri* individuals differed between the trials. This variation should most likely be interpreted as the result of behavioral plasticity or age‐related behavioral changes. However, our results show that relative differences in both locomotor activity and movement resumption after a simulated predator attack between individuals were maintained across trials. This implies the existence of consistent variation in behavior among individuals. Since behavioral responses and body size were not correlated in the studied fish we suggest that neurophysiological rather than biophysical differences underlie these observations, which is consistent with findings on zebrafish (*Danio rerio*) (Tran & Gerlai, 2013).

In personality research, behavioral traits are often found to be dependent of each other (Class & Brommer, 2015). Such behavioral correlations are however not unanimously supported in the literature (Garamszegi & Herczeg, 2012). Likewise, in this study we found only limited support for behavioral correlations. Behavioral correlations across different test setups could not be demonstrated. However, in the open field test, total distance traveled (as a measure of activity) was negatively correlated with the time spent in the centrum zone of the open field (as a measure of boldness). This result suggests that active individuals prefer the more risk‐averse peripheral zone, whereas less active individuals spend more time in the risk‐prone central zone in comparison to active individuals. As expected, time spent in the centrum zone was positively correlated with the number of times the fish entered the centrum. The absence of any further behavioral correlations should be subject to future research. We do remark however that the current study is not optimally designed to explore behavioral correlations, as such studies typically require larger sample sizes (Garamszegi & Herczeg, 2012).

The historical lack of interest in between‐individual behavioral variation has been used as justification to launch the field of personality research (Beekman & Jordan, 2017). However, this notion recently received criticism. It can be argued that individual behavioral variation was already the implicit corner‐stone of behavioral ecology—a discipline that explains animal behavior from a functional and evolutionary perspective (Beekman & Jordan, 2017; Owens, 2006). Moreover, the bulk of animal personality literature is descriptive rather than hypothesis‐driven and often lacks an evolutionary context and insight into the mechanistic underpinnings of behavioral variation (Beekman & Jordan, 2017; Roche et al., 2016; Sih, 2013). In light of the task ahead, the use of model organisms could be indispensable to bridge descriptive and hypothesis‐driven research, as suggested by Owens (2006). Behavioral ecologists typically study behavior of wild organisms rather than that of model organisms (Monaghan, 2014; Owens, 2006). Owens (2006) reports that <2% of the studies published in three leading journals in behavioral ecology between 2001 and 2005 made use of traditional model organisms. Likewise, Monaghan (2014) reports that in 2007–2014 less than 0.5% of the papers published in *Behavioral Ecology* made use of traditional model organisms. This is partly attributed to a general lack of knowledge on the natural history of traditional model organisms which has, in turn, been largely ascribed to an unfortunate division between biomedical sciences and eco‐evolutionary research (Alfred & Baldwin, 2015; Owens, 2006; Parichy, 2015). Consequently, behavioral ecologists miss out on a large amount of biochemical and physiological data that is available for traditional model organisms and fail to effectively link individual behavioral variation to the genetic and physiological mechanisms that underpin this variation (Beekman & Jordan, 2017; Owens, 2006). Although (field) studies on natural populations are of crucial importance, model organisms are under‐used in behavioral ecology. This has, in our opinion, contributed to the predominantly descriptive nature of animal personality research. The use of model organisms in combination with natural populations could extend the scope from descriptive studies to targeted quantitative research designs. In addition, it would also solve the lack of replication across laboratories that is common in behavioral ecology and help to validate findings, adding to the scrutiny of the field (Owens, 2006).

*Nothobranchius furzeri* is already a commonly used model organism in a range of biomedical research fields, in ecology and in evolutionary biology (Polačik et al., 2016). We argue that *N. furzeri* has great potential to further add to the advancement of ethology and behavioral ecology. Whereas personality variation has been reported in a range of traditional model organisms such as zebrafish (Tran & Gerlai, 2013), stickleback (Jolles, Taylor, & Manica, 2016), and rainbow trout (Elias, Thrower, & Nichols, 2018), this is the first study to report on personality variation in *N. furzeri*.

The main advantage of *N. furzeri* is its extremely short maturation time (3 weeks) and lifespan (typically <6 months). This is much shorter than that of traditional model organisms such as zebrafish with a typical maturation time of 2 months and lifespan of up to 5 years (Lawrence et al., 2012). In addition, the life history of the species is well characterized across ecological gradients (e.g. aridity, predation) and ongoing ecological and evolutionary research continuously adds to this knowledge (Blažek et al., 2016; Watters, 2009). This facilitates field studies to further elucidate the ecological causes and consequences of behavioral variation (Mittelbach et al., 2014). In complement with this ecological information, a whole brain atlas (D'angelo, 2013) along with an annotated genome (Reichwald et al., 2015; Valenzano et al., 2015) and transcriptome (Di Cicco et al., 2011) allow for an in‐depth investigation of neurophysiological and genetic underpinnings of behavioral variation. Moreover, successful transgenesis and the generation of transgenic lines (Hartmann & Englert, 2012) allows for the construction of specific lines. If such lines are characterized by different behavioral profiles, the molecular mechanisms underlying behavioral variation and the ecological and evolutionary implications of personality variation could be studied under well‐controlled experimental conditions (Tran & Gerlai, 2013).

Whereas studying the development of animal personality across ontogeny is pivotal to our understanding of the proximal causation, function (adaptive value), and evolution of personality, this remains largely understudied. Understanding the development of animal personality typically requires time‐consuming research as behavioral changes across the lifespan of individuals need to be monitored (Stamps & Groothuis, 2010). In this regard, the short lifespan of *N. furzeri* offers a major advantage over other vertebrate species. Moreover, its fast life cycle allows for time‐ and cost‐efficient examination of environmental underpinnings of behavioral variation across ontogeny—including experiential factors—and multigenerational setups.

While in the current study we identified stable individual differences in behavioral expression, thereby adding necessary fundamental information to the descriptive animal personality literature, we also provide a potential stepping stone to quantitative research designs by introducing the model organism *N. furzeri*. The inclusion of *N. furzeri* in behavioral sciences answers the call for an increased diversity in studied organisms (Monaghan, 2014). Its well‐characterized biomedical and ecological background in combination with its short life cycle make it an excellent model organism to further elucidate the causes and implications of behavioral variation in an eco‐evolutionary context.

## CONFLICT OF INTEREST

None declared.

## AUTHOR CONTRIBUTIONS

The study was designed by ESJT and TP and performed by ESJT and LS. Data was analyzed by ESJT. The manuscript was written by ESJT and reviewed by TP, LS, CP, and LB. All authors gave final approval for publication.

## DATA ACCESSIBILITY

Data is accessible at the FigShare repository (https://doi.org/10.6084/m9.figshare.6741185.v1).
